# Prediction of Neighbor-Dependent Microbial Interactions From Limited Population Data

**DOI:** 10.3389/fmicb.2019.03049

**Published:** 2020-01-21

**Authors:** Joon-Yong Lee, Shin Haruta, Souichiro Kato, Hans C. Bernstein, Stephen R. Lindemann, Dong-Yup Lee, Jim K. Fredrickson, Hyun-Seob Song

**Affiliations:** ^1^Biological Sciences Division, Pacific Northwest National Laboratory, Richland, WA, United States; ^2^Department of Biological Sciences, Tokyo Metropolitan University, Hachioji, Japan; ^3^National Institute of Advanced Industrial Science and Technology, Sapporo, Japan; ^4^Faculty of Biosciences, Fisheries and Economics, UiT – The Arctic University of Norway, Tromsø, Norway; ^5^The Arctic Centre for Sustainable Energy, UiT – The Arctic University of Norway, Tromsø, Norway; ^6^Whistler Center for Carbohydrate Research, Department of Food Science, Purdue University, West Lafayette, IN, United States; ^7^Bioprocessing Technology Institute, Agency for Science, Technology and Research, Singapore, Singapore; ^8^School of Chemical Engineering, Sungkyunkwan University, Seoul, South Korea; ^9^Department of Biological Systems Engineering, University of Nebraska-Lincoln, Lincoln, NE, United States; ^10^Nebraska Food for Health Center, Department of Food Science and Technology, University of Nebraska-Lincoln, Lincoln, NE, United States

**Keywords:** microbial communities, microbial ecology, context dependence, network inference, interspecies interactions

## Abstract

Modulation of interspecies interactions by the presence of neighbor species is a key ecological factor that governs dynamics and function of microbial communities, yet the development of theoretical frameworks explicit for understanding context-dependent interactions are still nascent. In a recent study, we proposed a novel rule-based inference method termed the Minimal Interspecies Interaction Adjustment (MIIA) that predicts the reorganization of interaction networks in response to the addition of new species such that the modulation in interaction coefficients caused by additional members is minimal. While the theoretical basis of MIIA was established through the previous work by assuming the full availability of species abundance data in axenic, binary, and complex communities, its extension to actual microbial ecology can be highly constrained in cases that species have not been cultured axenically (e.g., due to their inability to grow in the absence of specific partnerships) because binary interaction coefficients – basic parameters required for implementing the MIIA – are inestimable without axenic and binary population data. Thus, here we present an alternative formulation based on the following two central ideas. First, in the case where only data from axenic cultures are unavailable, we remove axenic populations from governing equations through appropriate scaling. This allows us to predict neighbor-dependent interactions in a *relative* sense (i.e., fractional change of interactions between with versus without neighbors). Second, in the case where both axenic and binary populations are missing, we parameterize binary interaction coefficients to determine their values through a sensitivity analysis. Through the case study of two microbial communities with distinct characteristics and complexity (i.e., a three-member community where all members can grow independently, and a four-member community that contains member species whose growth is dependent on other species), we demonstrated that despite data limitation, the proposed new formulation was able to successfully predict interspecies interactions that are consistent with experimentally derived results. Therefore, this technical advancement enhances our ability to predict context-dependent interspecies interactions in a broad range of microbial systems without being limited to specific growth conditions as a pre-requisite.

## Introduction

The interactions between microorganisms often dictate community-level functions and contributions to many biogeochemical and ecosystem processes. Microbes interact with plants, animals, and humans directly by building relationships with hosts (Bordenstein and Theis, 2015; Hassani et al., 2018), and/or by indirectly controlling the natural cycle of chemical elements that make up living organisms (Falkowski et al., 2008; Kirchman, 2018). Microbes perform these essential functions not individually, but as communities of species that help or compete with each other. Partnerships among members are typically dynamic and can vary in response to environmental cues, which gives rise to the concept of *context-dependent interaction*. A fundamental understanding of context dependence has been elusive to microbial ecologists because the way interactions occur in most natural communities is typically too complex to untangle. Thus, new theoretical frameworks that can use tractable amounts of experientially derived measurements are the key for the predicting context-dependent interactions, yet advancements in this area remain nascent.

In principle, context-dependent interactions may be examined by existing network inference methods (Song et al., 2014). For example, the correlative relationships of species populations across different environmental conditions or community memberships generate distinct interspecies interaction networks, the comparison of which may provide an idea of how interactions are modulated by the impact of abiotic and biotic factors. The success of this comparative analysis largely relies on robust, accurate predictions of interaction networks and their reorganization across conditions. However, comprehensive data are rarely available for most of the microbial ecosystems that are studied. Even for simple cases where context-dependent interactions are not an issue, inference results are often inconsistent among different similarity metrics (Faust and Raes, 2012). In a test using time-series community data, correlation-based methods were also shown ineffective in inferring microbial interactions (Coenen and Weitz, 2018). Aside from such technical issues, correlation-based approaches do not provide a fundamental understanding of how interspecies interactions are modulated by dynamic environments and/or the presence or absence of specific partners.

In a recent study, we proposed a computational approach to predict how microbial interactions can be modulated by the addition of new members to the community in ecological systems (Song et al., 2019). Taking pairwise interactions in binary communities as a basis, the approach enables predicting the change in interactions in the presence of new members by assuming that the resulting shifts will be minimal and is thus termed Minimal Interspecies Interaction Adjustment (MIIA). The resulting prediction showed a fairly good robustness against noise in population data for complex communities. In this initial development, however, the predictive capability of MIIA was evaluated under conditions where all species can grow independently and within binary partnerships. While useful for the conceptual development, these conditions may be too strict to cover symbiotic relationships including syntrophic interactions between bacterial species which have been widely observed in microbial communities in natural environments (Kouzuma et al., 2015). For instance, mutual metabolic dependence of fatty acids oxidizing bacteria and methanogens make them unable to grow independently, but able to grow together as a community (Kato and Watanabe, 2010). In these cases where organisms show growth dependence on each other, axenic and binary population data are not fully available, which can be problematic for the MIIA approach because it becomes impossible to identify binary interaction coefficients.

To overcome this limitation, we present an alternative formulation and expansion of the MIIA approach so that it can account for interspecies growth dependencies without being constrained by the lack of the full availability of axenic and binary culture data. Two key ideas presented herein include: (1) model scaling (i.e., reformulation of model equations to remove the effects of monoculture data) and (2) determination of unknown interaction parameters through parameter sensitivity analysis. Model scaling enables estimating binary interaction coefficients even in the absence of axenic populations, if binary growth data are available. Parameter sensitivity is required when binary interaction coefficients are inestimable even with model scaling due to the absence of both axenic and binary growth data. Through case studies, we demonstrated how this new formulation can reliably predict neighbor-dependent interactions. Overall, our predictions showed a fairly good agreement with experimental understanding, while additional experimental analyses were required when no data is available to estimate binary interaction coefficients. The proposed method can guide new experimental designs in this regard. This enhanced approach for predicting context-dependent microbial interactions demonstrates a significant extension of previous approaches and provides means to evaluate a wider range of microbial systems, offering itself as a practically useful tool for studying synthetic and natural microbial communities.

## Materials and Methods

### MIIA in a Nutshell

MIIA predicts pairwise interactions in multi-species communities through the following two steps: (1) estimation of interaction coefficients in binary cultures, and (2) prediction of the shifts in interactions by additional members based on a minimal adjustment hypothesis.

(1) $$\frac{x_{i}^{B}-x_{i}^{A}}{x_{i}^{A}}=a_{i,j}^{B}\,x_{j}^{B}$$

(2) $$\frac{x_{i}^{C}-x_{i}^{A}}{x_{i}^{A}}=\sum_{j=1,\neq i}^{N}a_{i,j}^{C}\,x_{j}^{C}$$

**FIGURE 1:**
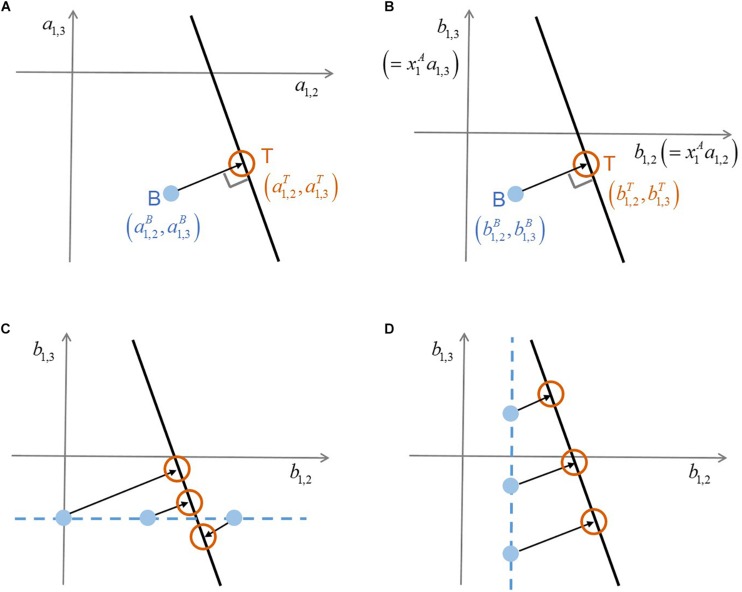
A schematic illustration of the conceptual difference between original MIIA vs. the proposed approach that combines model scaling and sensitivity analysis. **(A)** Prediction of interaction coefficients in a ternary community $\left(a_{1,2}^{T},a_{1,3}^{T}\right)$ through the orthogonal projection of binary interaction coefficients $\left(a_{1,2}^{B},a_{1,3}^{B}\right)$, **(B)** consistent orthogonality between the two vectors of interaction coefficients on the scaled coordinates where $\left(b_{1,2}^{T},b_{1,3}^{T}\right)=\left(x_{1}^{A}\,a_{1,2}^{T},x_{1}^{A}\,a_{1,3}^{T}\right)$ and $\left(b_{1,2}^{B},b_{1,3}^{B}\right)=\left(x_{1}^{A}\,a_{1,2}^{B},x_{1}^{A}\,a_{1,3}^{B}\right)$, **(C)** sensitivity of predicted interaction coefficients to the variation of $b_{1,2}^{B}$, **(D)** sensitivity of predicted interaction coefficients to the variation of $b_{1,3}^{B}$. Superscripts *A*, *B*, and *T* denote ‘axenic’, ‘binary’, and ‘ternary’ cultures, respectively.

• **Interactions in binary communities:** Song et al. (2019) used the following formula that estimates the effect of species *j* on *i* in the binary culture (denoted by $a_{i,j}^{B}$):
  where the superscripts ‘A’ and ‘B’ represent ‘axenic’ and ‘binary’ cultures, respectively, and *x*_*i*_ and *x*_*j*_ denote the population densities (i.e., abundance) of species *i* and *j* in stagnant phase (i.e., at the end of growth phase) or in steady state. Song et al. (2019) also showed that Equation (1) can be derivable from a generalized Lotka–Volterra model (Wangersky, 1978) under steady-state condition. The left-hand side of Equation (1) represents per capita interaction strength (Paine, 1992).
• **Shifts in interactions by additional members:** The binary culture model given in Equation (1) is extended to multi-species communities as follows:
  where the superscript ‘C’ denotes ‘complex’ communities (that include more than two species) and *N* indicates the number of species including additional members. Interaction coefficients cannot be uniquely determined from this single equation that contains (*N*-1) unknowns (i.e., $a_{i,j}^{C}$’s). In this case, there exist infinite solutions that satisfy Equation (2), which form a hyperplane in the (*N*-1)-dimensional space of interaction coefficients ($a_{i,j}^{C},i\neq j$). Based on the assumption that the adjustment of binary interaction coefficients by additional members (i.e., measured by Euclidean distance between $a_{i,j}^{B}$’s and $a_{i,j}^{C}$’s) will be minimal, MIIA predicts the vector $a_{i,j}^{C}$’s as the point on the hyperplane closest to the vector of $a_{i,j}^{B}$’s. This solution is simply obtained by orthogonal projection of $a_{i,j}^{B}$’s on the (*N-1*)-dimensional hyperspace formed by Equation (2). Figure 1A illustrates how such orthogonal projection occurs for the case of a three-member community. Modulation of pairwise interactions by introducing new neighbors can be quantified based on the difference between $a_{i,j}^{B}$ and $a_{i,j}^{C}$. As a special case, if the point of $a_{i,j}^{B}$’s happens to be on the hyperplane, no modulation is predicted to occur by additional members (because the distance of interaction coefficients between binary and complex communities is zero). For more details, the original paper by Song et al. (2019) should be referred to.

### Model Scaling

Species that have strong dependencies on partners may not grow independently. Axenic population densities in this case can be unobtainable, which limits the originally described MIIA approach. In Equation (1), the values of $a_{i,j}^{B}$ may explode for a very low axenic density of species *i* (i.e., $x_{i}^{A}\ll 1$) and also for a low population of its binary partner, i.e., $x_{j}^{B}\ll 1$; becomes unidentifiable if the values of $x_{i}^{A}$ and $x_{j}^{B}$ are unavailable. In this section, we provide an idea of handling the case where the value of $x_{i}^{A}$ is extremely low or unavailable; in the next section, we will consider the case where the value of $x_{j}^{B}$ is extremely low or unavailable.

Without compromising generality, we reformulated the model equations for binary and complex communities by multiplying $x_{i}^{A}$ on both sides of Equations (1) and (2), which results in

(3) $$x_{i}^{B}-x_{i}^{A}=b_{i,j}^{B}\,x_{j}^{B}$$

(4) $$x_{i}^{C}-x_{i}^{A}=\sum_{j=1,\neq i}^{S}b_{i,j}^{C}\,x_{j}^{C}$$

where $b_{i,j}^{B}$ and $b_{i,j}^{C}$ are interaction coefficients scaled by $x_{i}^{A}$, i.e.,

(5) $$b_{i,j}^{B}\equiv x_{i}^{A}\,a_{i,j}^{B}$$

(6) $$b_{i,j}^{C}\equiv x_{i}^{A}\,a_{i,j}^{C}$$

The scaling above is always possible for any non-zero value of $x_{i}^{A}$. Thus, it implies that we translate ‘no growth’ as a minor presence – i.e., below the limit of detection – in place of absolute absence. The minimal adjustment rule of MIIA still applies to Equations (3) and (4) because the orthogonal relationship between binary and complex interaction coefficients established in the original space, remains valid on the new coordinates rescaled with a constant factor $x_{i}^{A}$ (Figure 1B). We termed this extension *scaled* MIIA (s-MIIA).

The quantitative values of interaction coefficients predicted by the s-MIIA are different from those by the original MIIA. That is, what is determined with the s-MIIA is ‘scaled’ interaction coefficients (i.e., $b_{i,j}^{B}$ and $b_{i,j}^{C}$), rather than ‘absolute’ values (i.e., $a_{i,j}^{B}$ and $a_{i,j}^{C}$). However, it should be noted that the resulting scaled coefficients can also provide sufficient information required to predict how interaction changes in response to the addition of new neighbors, a primary question the MIIA aims to address. Thus, when the scope of prediction is confined to ‘relative’ interaction changes, both the original version and the s-MIIA generate the same result, i.e.,

(7) $$\Delta \,b_{i,j}^{r\,e\,l,B\to C}\equiv \frac{b_{i,j}^{C}-b_{i,j}^{B}}{b_{i,j}^{B}}=\frac{a_{i,j}^{C}-a_{i,j}^{B}}{a_{i,j}^{B}}\equiv \Delta \,a_{i,j}^{r\,e\,l,B\to C}$$

or

(8) $$\Delta \,b_{i,j}^{r\,e\,l,C\to B}\equiv \frac{b_{i,j}^{B}-b_{i,j}^{C}}{b_{i,j}^{C}}=\frac{a_{i,j}^{B}-a_{i,j}^{C}}{a_{i,j}^{C}}\equiv \Delta \,a_{i,j}^{r\,e\,l,C\to B}$$

where $\Delta \,a_{i,j}^{r\,e\,l}$ and $\Delta \,b_{i,j}^{r\,e\,l}$ denote the relative changes in interaction coefficients *a*_*i,j*_ and *b*_*i,j*_ that are predicted by the original and scaled MIIA, respectively. The superscripts *B*→*C* and *C*→*B* denote the interaction changes from binary to complex cultures and from complex to binary cultures, respectively. From Equations (7) and (8), therefore, it is clear that the predictions by the original formulation and the s-MIIA are exactly the same with respect to the relative changes in interaction coefficients.

### Estimation of Binary Interaction Coefficients Using a Sensitivity Analysis

While the scaling method above resolves the issue associated with extremely low or non-measurable axenic populations, identifying binary interaction coefficients is also dependent on the availability of binary co-culture data. We considered eight different growth scenarios in axenic and binary cultures and showed when binary interaction coefficients are identifiable by the original and scaled MIIA and when not (Table 1). For Cases I and II, binary interaction coefficients can be estimated either by the original formulation or the scaling method. Cases V and VI highlight the situations that could not be handled by the original formulation, but only by the scaling method. The remaining cases (i.e., Cases III, IV, VII, and VIII) are challenging scenarios, to which neither the original nor scaling method can be naively applied. However, we exclude Cases III and VIII from our consideration because these events might be rare if at all possible; therefore, our focus for demonstration purposes is placed on Cases IV and VII.

**TABLE 1:** Comparison between the original MIIA and the model scaling in regard to the estimation of binary interaction coefficients in various possible growth scenarios in axenic and binary cultures.

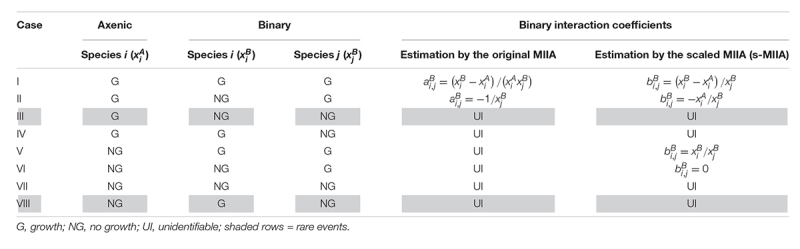

In order to estimate binary interaction coefficients for Cases IV and VII, we take $b_{i,j}^{B}$ as an adjustable model parameter with an aim to determining its rational value or range based on the sensitivity analysis and the comparison with experimental evidence. The success of this parameterization strategy depends on several factors: (1) what binary interaction coefficients are unidentifiable, (2) what the complex community model [i.e., Equation (2)] looks like, and (3) the availability of experimental evidence to determine ranges of parameters. Figures 1C,D illustrate how the first and second factors affect predictions by considering the following two scenarios: (1) $b_{1,3}^{B}$ were determined but not $b_{1,2}^{B}$, and (2) $b_{1,2}^{B}$ was determined but not $b_{1,3}^{B}$. In the respective case, $b_{1,3}^{B}$ and $b_{1,2}^{B}$ are chosen as adjustable parameters. In the hypothetical scenario in Figures 1C,D, the prediction of interaction coefficients in the ternary community $b_{1,2}^{T}$ and $b_{1,3}^{T}$ is relatively less sensitive to the variation of the parameter $b_{1,2}^{B}$ in comparison to $b_{1,3}^{B}$. This distinct sensitivity with respect to $b_{1,2}^{B}$ and $b_{1,3}^{B}$ of course depends on the slope of the linear ternary community model (*l*^*T*^). As such, the sensitivity analysis evaluates the robustness of predictions and specifically shows which predictions are sensitive and which need additional data to reduce uncertainty.

### Calculation of Variable Ratios From Multiplicate Data

Estimation of binary interaction coefficients in MIIA (both original and new formulations) (i.e., $a_{i,j}^{B}$’s and $b_{i,j}^{B}$’s) include the ratio of variables as shown in Equations (1) and (3). A caution is needed in calculating them from multiplicate data because the mean value of ratio can be different from the ratio of means. This so-called bias issue is fundamentally associated with the small sample size (Cochran, 1977) and often arises in biological experiments where the sample size is typically limited to 3 to 5. To evaluate the impact of the variability in small-size samples on the ratio estimation for the datasets used in our case studies, we first accounted for all possible combinations of three replicates of each variable, e.g., 27 combinations from three variables ($x_{i}^{A}$, $x_{i}^{B}$, and $x_{j}^{B}$) and then compared estimated values of $a_{i,j}^{B}$’s and $b_{i,j}^{B}$ between the following two cases: (1) taking the mean of ratio and (2) taking the ratio of means. For the Wang et al.’ (2017) data used in the first case study (that shows relatively more significant variation than the data of the second case study), we observed only negligible differences between the two cases, i.e., <1.4% for $b_{i,j}^{B}$; <2.8% for $a_{i,j}^{B}$.

## Results

To demonstrate how the MIIA can predict membership-dependent interactions despite limited population data, we analyzed two experimental datasets taken from published literature: (1) three-member species derived from natural microbial community in a paddy soil (Wang et al., 2017) and (2) four-member species isolated from a cellulose-degrading bacterial community enriched from a composting process (Kato et al., 2008). The first example represents the ideal case (corresponding to Case 1 in Table 1) when all organisms can grow both in axenic and binary cultures, as well as the ternary culture. This enables MIIA to use the full estimation of binary interaction coefficients. We chose this dataset to demonstrate the effectiveness of the proposed model scaling method through the comparison with the original formulation. In the second example, we extend the analysis to a more challenging – yet realistic – situation where some of the organisms cannot grow in axenic and binary cultures. In this case, the original MIIA becomes ineffective because only a subset of binary interaction coefficients can be estimated, requiring the new method proposed in this work.

### Evaluation of the Scaling Method Using a Bacterial Community for Which All the Binary Interaction Coefficients Are Estimable

The study published from Wang et al. (2017) provides population data for three soil organisms that are known to grow in all combinations of axenic, binary and ternary cultures. The three bacterial species analyzed in their work include: *Leuconostoc lactis* (LL), *Janthinobacterium lividum* (JL), and *Lactococcus piscium* (LP). The copy number of the 16S rRNA genes – as determined by quantitative PCR – has been used as a proxy for the density of each species (see Supplementary Table S1 for the raw data retrieved from the original paper). We used this consortium data as a technical proof-of-concept example to illustrate how the scaled MIIA works and how to interpret its predictions.

**FIGURE 2:**
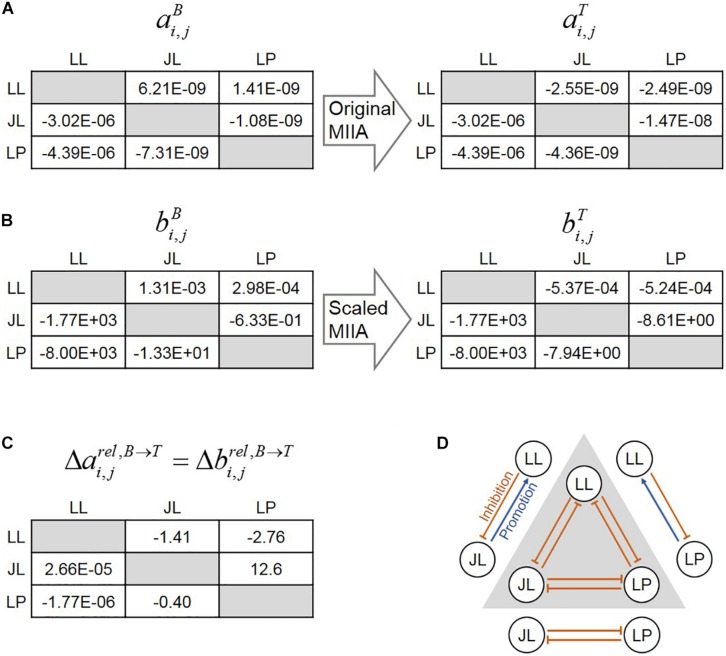
Shifts in interspecies interactions in binary and ternary cultures for the soil microbial consortium studied by Wang et al. (2017). **(A)** prediction of interaction coefficients (*a*_*i,j*_’s) by the original MIIA for binary (left) and ternary (right) cultures. **(B)** Prediction of interaction coefficients (*b*_*i,j*_’s) by the scaled method for binary (left) and ternary (right) cultures. In **(A,B)**, the numerical value in the (*i, j*) entry of the matrices on the left and right denotes the estimation of *a*_*i,j*_ or *b*_*i,j*_ (i.e., the effect of species *j* on *i*) in the binary and ternary cultures. Differences in interaction coefficients of the two matrices on the left and right represents the predicted changes in pairwise interactions by the addition of a new member. **(C)** Relative changes in interaction coefficients predicted either from the original MIIA ($\Delta \,a_{i,j}^{r\,e\,l,B\to T}$) or the scaled method ($\Delta \,b_{i,j}^{r\,e\,l,B\to T}$). **(D)** Graphical representation of context-dependent interaction predicted either from the original MIIA or the scaled method (arrows outside and inside the triangle denote interactions in binary and ternary communities). In *a*_*i,j*_ and *b*_*i,j*_, *i, j* ∈{*LL*, *JL*, *LP*}.

The original MIIA predicted absolute interaction coefficients in binary and ternary communities ($a_{i,j}^{B}$ and $a_{i,j}^{T}$) (Figure 2A), while the s-MIIA estimated their scaled values ($a_{i,j}^{B}\,x_{i}^{A}$ and $a_{i,j}^{T}\,x_{i}^{A}$, i.e., $b_{i,j}^{B}$ and $b_{i,j}^{T}$) (Figure 2B). Both methods predicted sign changes for some cases: interaction coefficients *a*_*LL,JL*_ and *a*_*LL,LP*_ were positive (i.e., promotive) in binary cultures, but became negative (i.e., inhibitory) in the ternary culture. This prediction was consistent with the experimental observations in the original paper by Wang et al. (2017).

It should be noted that the s-MIIA still allows us to assess the changes in interactions in the ternary community because the same constant (i.e., $x_{i}^{A}$) was multiplied on both $a_{i,j}^{B}$ and $a_{i,j}^{T}$. As a critical difference from the original method, the prediction of the s-MIIA is limited to relative comparison of interaction parameters only for the same organism that is influenced by others. That is, we can compare *b*_*i,j*_ values across different *j*’s along each row in the matrix of interaction coefficients (Figure 2B), but not across different *i*’s because the values of the scaling constant $x_{i}^{A}$ are different among species. As expected, predicted relative changes of interactions coefficients – e.g., defined in Equation (7) – were identical between the original and scaled methods (Figure 2C). The plus and minus signs in the table of Figure 2C denote the change of interactions in the ternary community in positive and negative directions relative to interactions in binary communities. In Figure 2D, we illustrated how the relationships between two species can be changed in binary and ternary communities. Wang et al.’s (2017) data showed reasonably small standard deviations of population densities for both axenic and binary cultures (from 0.006 to 0.069) and the ternary culture (from 0.022 to 0.094) where standard deviations were calculated using a multiplicative lognormal noise function as described in Supplementary Table S1 as well as in the previous paper by Song et al. (2019). The measurement error in this range did not deteriorate the predictive power of s-MIIA (Supplementary Figure S1; see also Song et al., 2019).

### Analyzing Communities Composed of Species That Cannot Grow Independently

We extend our analysis to a more challenging case where the full estimation of binary interaction coefficients by the original MIIA is not possible due to the ineffective growth of some of the member species in axenic and binary cultures. These datasets were obtained from defined mixed cultures using four bacterial strains previously studied by Kato et al. (2008): a cellulose-degrading anaerobe (*Clostridium straminisolvens* CSK1), a saccharide-utilizing anaerobe (*Clostridium thermosuccinogenes* FG4), a peptide- and acetate-utilizing aerobe (*Pseudoxanthomonas taiwanensis* M1-3) and a peptide-, glucose-, and ethanol-utilizing aerobe (*Brevibacillus agri* M1-5), which are hereafter denoted by CS, CT, PT and BA, respectively. A number of different types of interactions such as trophic interactions, competition, and lethal inhibition as well as growth promotion have been detected in these bacterial communities (Kato et al., 2008; Yamamoto et al., 2010). When cultured together, these four organisms formed a stable consortium. With simpler memberships that do not contain all four members, however, some of the organisms could not grow as mentioned above. For instance, the growth of anaerobe CS requires the presence of aerobes (such as PT or BA) for the removal of oxygen; the anaerobe CT not only depends on aerobes, but also on CS that can degrade cellulose into saccharides. We summarized all interaction features below and provided the raw data retrieved from Kato et al. (2008) in Supplementary Table S2:

• In axenic cultures, PT and BA can grow, whereas neither CS nor CT can grow alone.
• Out of the six possible binary combinations among four species, two species can co-grow in the three pairs (CS-PT, CS-BA, and PT-BA); only one species can grow in the two pairs (CT-PT and CT-BA); none of them can grow in the CS-CT pair.
• Out of the four possible ternary combinations, all three species can co-grow in the three consortia (CS-CT-PT, CS-CT-BA, and CS-PT-BA), but only two species (PT and BA) can co-grow in the CT-PT-BA consortium.
• All four species can co-grow in the quaternary culture.

Strong growth dependency of CS and CT on other member species led to the absence of population data derived from axenic cultures and certain binary parings. Consequently, the original MIIA identified only 33% (4 out of 12) of the binary interaction coefficients (Supplementary Table S3). With this partial identification of interaction coefficients in binary communities, the original MIIA could not provide any predictions for ternary and quaternary communities except the CS-PT-BA consortium, the estimation of which was limited to 33% of interactions. The s-MIIA generated improved results by estimating 66% of the binary interaction (8 out of 12) and predicting 100% of interaction coefficients in the CS-PT-BA consortium. Prediction for other multi-species communities was limited however: only 16.7% in the CT-PT-BA consortium and no predictions for all other communities (Supplementary Table S3). This result shows that despite improvement, the scaling method alone is not sufficient to handle the cases where organisms cannot co-grow in binary communities (i.e., Cases IV and VII in Table 1), therefore requiring additional analyses to overcome this limitation.

### Sensitivity Analysis of Unidentifiable Interaction Parameters

While the scaling method provided expanded estimates of binary interaction coefficients in comparison to the original method, the datasets from Kato et al. (2008) still contain several binary interaction coefficients that remain unknown. These unknown binary coefficients ($b_{C\,S,C\,T}^{B}$, $b_{C\,T,C\,S}^{B}$, $b_{P\,T,C\,T}^{B}$, and $b_{B\,A,C\,T}^{B}$) are all associated with CT, which shows no growth in the axenic or even binary cultures. Our strategy to overcome this limitation is to take these four coefficients as adjustable parameters to examine how MIIA predictions would vary as their functions (see section Materials and Methods) and to determine their most plausible values or ranges based on the consistency between final model predictions with any experimental observations.

No direct experimental evidences were available from Kato et al. (2008) that can be used to decisively determine specific values of the four unknown binary parameters. However, they provided an interspecies interaction network for the four-member consortium that could be used as a basis to inform some of the unidentifiable binary interaction coefficients. This interaction network was derived from a combined analysis of three complementary datasets including those reported elsewhere (Kato et al., 2004, 2005; Yamamoto et al., 2010). To summarize their integrative analysis of experimental data: (1) they analyzed substrate utilization profiles and metabolites of each member under axenic culture conditions to propose exchange scenarios among the members; (2) they examined how the growth of a member in pure cultures can be promoted or suppressed when a cell-free culture filtrate of another member was added to the growth media; (3) they compared populations of a member (e.g., CS) between in the presence and the absence of another member (e.g., PT) to examine the effects of one on others (in the above example, effects of PT on the population of CS).

**FIGURE 3:**
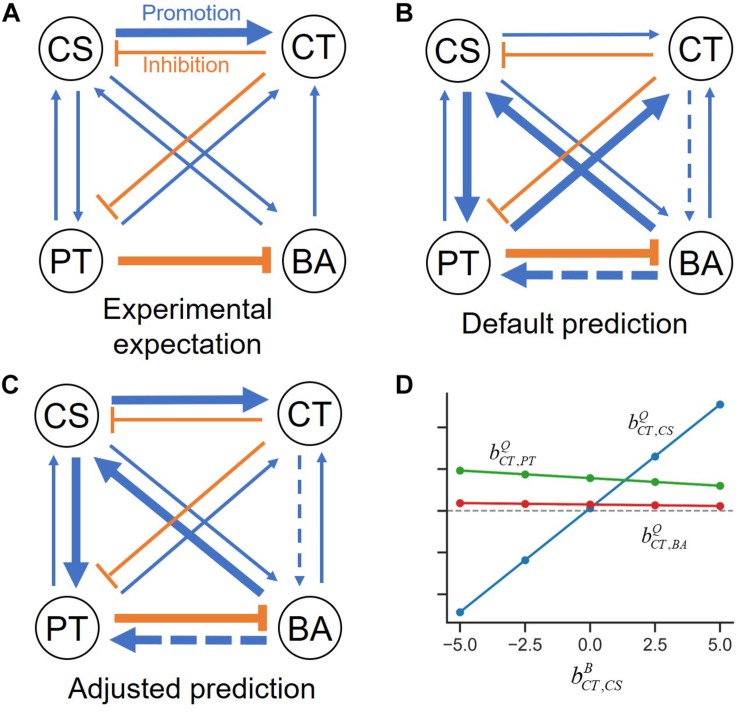
Interspecies interaction networks in the quaternary community. **(A)** Interaction network derived from the data analysis by Kato et al. (2008), **(B)** predicted interaction network by setting four binary interaction parameters zeros, **(C)** refined network by adjusting the value of $b_{C\,T,C\,S}^{B}$ to 2.5 such that the effect of CS on CT becomes relatively stronger, **(D)** the variation of interaction coefficients in the quaternary community ($b_{C\,T,C\,S}^{Q}$,$b_{C\,T,P\,T}^{Q}$, and $b_{C\,T,B\,A}^{Q}$) to the change of $b_{C\,T,C\,S}^{B}$, which provides justification for adjusting $b_{C\,T,C\,S}^{B}$.

An overall interspecies interaction network among the four species depicted by Kato et al. (2008) show various types of interspecies interactions among member species (Figure 3A). For demonstration purpose, we took this network as a reliable specific interaction scenario, while it may not represent true interactions in the quaternary community. Figure 3B is the prediction of the s-MIIA obtained based on default parameters, $b_{C\,S,C\,T}^{B}=b_{C\,T,C\,S}^{B}=b_{P\,T,C\,T}^{B}=b_{B\,A,C\,T}^{B}=0$, i.e., by assuming neutral interactions for unknown interactions in binary communities. Surprisingly, this default prediction showed no contradiction with the interaction network experimentally derived by Kato et al. with respect to the positive and negative effects among four species although providing two additional interactions (dotted lines in Figure 3B) that were not seen in Figure 3A. As to the magnitude of interactions, however, there were some discrepancies between the two networks. In particular, Kato et al. identified two stronger interactions, i.e., the promotive effect of CS on CT and inhibitive effect of PT on BA (Figure 3A), the former of which was however not captured in the default network (Figure 3B). As addressed in the previous section, the comparison of interactions predicted by the s-MIIA is relative and therefore limited to the effects of different species for the same species that is influenced. With this limitation in mind, we tried to adjust unknown binary interaction parameters so that interaction coefficients in the quaternary community are consistent with the experimentally derived network. In this case, we were able to obtain a stronger effect of CS on CT in the quaternary network by increasing the value of $b_{C\,T,C\,S}^{B}$ to 2.5 based on the parameter sensitivity analysis. The resulting interaction network and sensitivity profiles were shown in Figures 3C,D.

**FIGURE 4:**
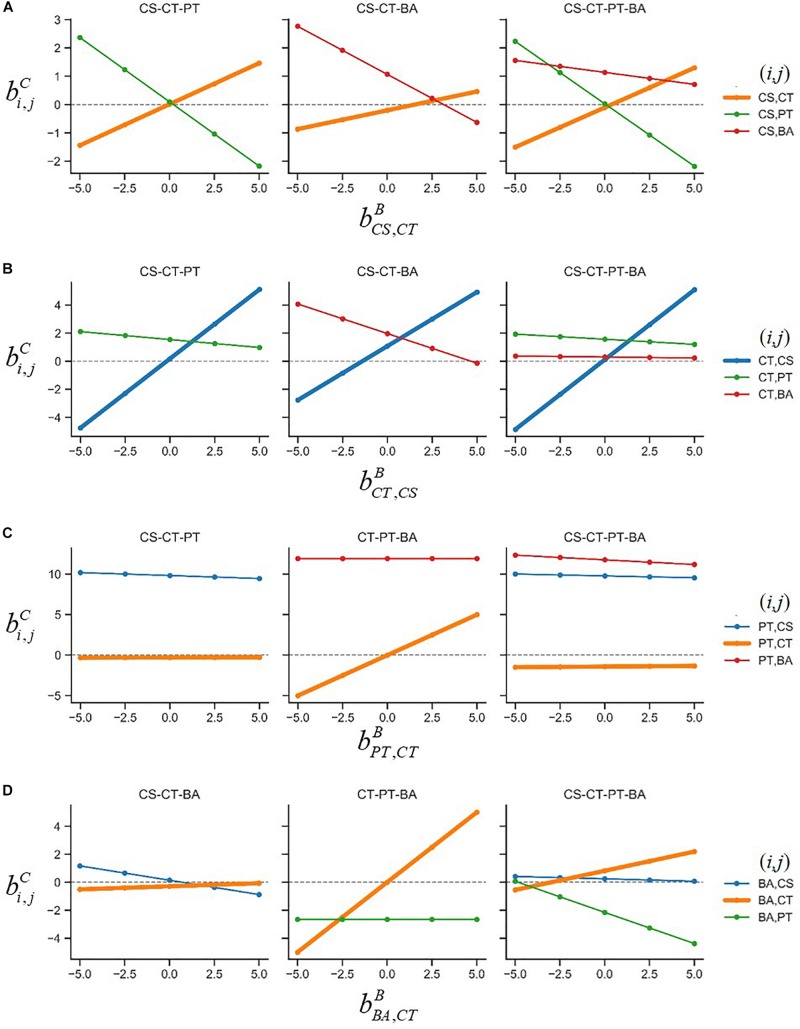
Sensitivity of interaction coefficients predicted for ternary and quaternary communities $b_{i,j}^{C}$ against the variation of assumed binary interaction parameters: **(A)** $b_{C\,S,C\,T}^{B}$, **(B)** $b_{C\,T,C\,S}^{B}$, **(C)** $b_{P\,T,C\,T}^{B}$, and **(D)** $b_{B\,A,C\,T}^{B}$. The coefficient $b_{i,j}^{C}$ means the effect of species *j* on species *i*, and the superscript C represents complex communities composed of three- or four-member species as denoted on the top of each panel. The (*i, j*) pairs were provided to the right of each row.

The sensitivity analysis was extended to all parameters (Figure 4). Interaction coefficients in ternary and quaternary cultures that show weak dependency on binary interaction parameters indicate that these insensitive coefficients can be robustly predicted regardless of assumed binary parameter values. Similarly, this implies that the process for determining interaction coefficients is sensitive to the assumed binary parameters (including those that may change their signs) and that additional experimental understanding on interactions in ternary and/or quaternary cultures can be required.

### Robust Predictions Despite Parameter Uncertainties

While an appropriate value for $b_{C\,T,C\,S}^{B}$ was determined by integrating the parameter sensitivity analysis result and the experimental understanding from Kato et al., we lack further data or experimental observations that can be used to determine other parameters, i.e., $b_{C\,S,C\,T}^{B}$, $b_{P\,T,C\,T}^{B}$, and $b_{B\,A,C\,T}^{B}$. In order to examine the effects of the choice of these parameters, we considered the following three cases:

• Case 1: $b_{C\,S,C\,T}^{B}=b_{C\,T,C\,S}^{B}=b_{P\,T,C\,T}^{B}=b_{B\,A,C\,T}^{B}=0$
• Case 2: $b_{C\,T,C\,S}^{B}=2.5$ and $b_{C\,S,C\,T}^{B}=b_{P\,T,C\,T}^{B}=b_{B\,A,C\,T}^{B}=0$
• Case 3: $b_{C\,T,C\,S}^{B}=2.5$, $b_{C\,S,C\,T}^{B}=-0.5$, $b_{P\,T,C\,T}^{B}=0.1$, and $b_{B\,A,C\,T}^{B}=2.5$

As mentioned previously, Case 1 denotes default setting that assumes neutral interactions; Case 2 is a simple adjustment of a minimum number of parameters (i.e., $b_{C\,T,C\,S}^{B}$) in order to match with experimental understanding. Case 3 represents an example of alternative parameter setting that leads to the same quaternary network as in Case 2, but different predictions in ternary networks.

**FIGURE 5:**
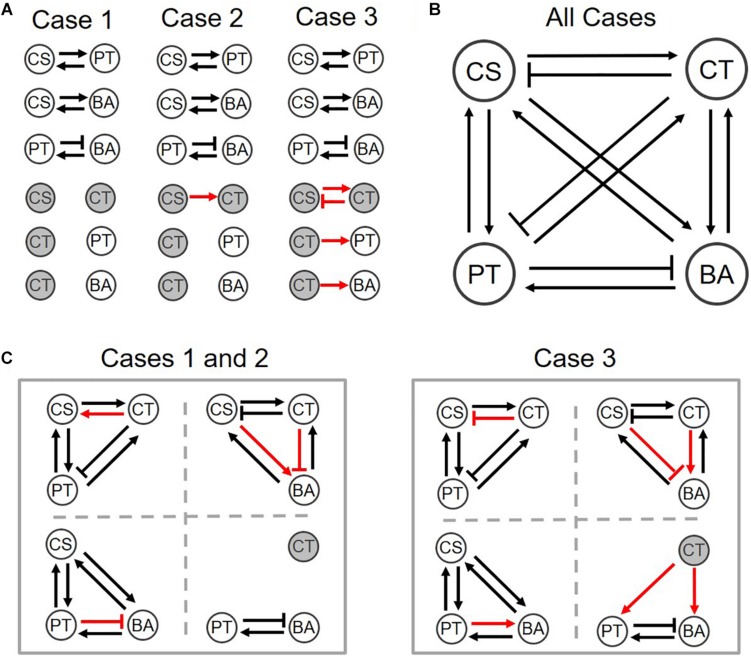
Interspecies interaction networks for **(A)** binary, **(B)** quaternary, and **(C)** ternary communities of three different parameter settings. Normal arrows and bar arrows describe the positive and negative influences, respectively. Black and red colors indicate the case-independent and -dependent interactions. Hollow and filled circles respectively indicate if species can co-grow in the communities or not. See the detail values in Supplementary Table S4.

We provided graphical representations of binary interactions for the three cases described above (Figure 5A). These three scenarios equally predict the structure of the interaction network in the quaternary community experimentally determined by Kato et al. (Figure 5B). Despite variations of assumed binary coefficients among three cases, predicted interactions in ternary communities were fairly robust. Particularly, Cases 1 and 2 generated exactly the same ternary interaction networks (left panel of Figure 5C). Overall, we found that out of 24 possible interactions in ternary communities, 75% of interactions were consistently predicted across three different parameter settings, while 25% predictions were not. When binary and quaternary communities were included, the portion of robust predictions increased to 79% (i.e., 38 out of the 48 interactions in total of binary, ternary, and quaternary communities). Therefore, these results (including both case-independent and case-specific predictions) can be used to inform the design of new experiments for further validation.

### Microbial Interactions Changing Across Different Partnerships

**FIGURE 6:**
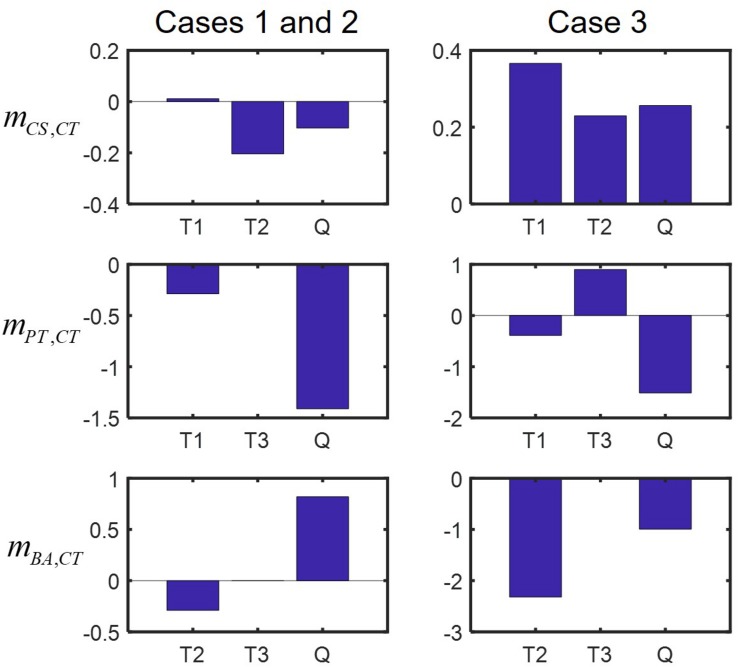
Predicted modulation of interactions in binary and complex communities quantified by $b_{i,j}^{C}-b_{i,j}^{B}\;\left(\equiv m_{i,j}\right)$, a metric used in Song et al. (2019). Plus (or minus) sign of *m*_*i,j*_ does not necessarily mean that the sign change in interactions occurs, but implies that the influence of species *j* on *i* is shifted in a positive (negative) direction in the presence of new neighbors: left panels: Cases 1 and 2, right panels: Case 3. T1 = CS-CT-PT community, T2 = CS-CT-BA community, T3 = CS-PT-BA community, and Q = CS-CT-PT-BA community.

We finally examined how interspecies interactions could be modulated by the addition of new members or the loss of existing members. Interaction networks predicted across binary, ternary and quaternary cultures (Figure 5) showed that interspecies interactions can be membership dependent, while specific predictions were case-dependent. For example, predictions for Cases 1 and 2 showed that CS and CT, who could not coexist in the binary culture (Figure 5A), exhibit mutualism in the presence of PT, but show antagonism in the presence of BA (the left panel of Figure 5C) or both PT and BA (Figure 5B). By contrast, Case 3 predicted no such shifts, i.e., the relationship of CS and CT remained antagonistic regardless of who are their neighbors (the right panels of Figures 5A–C). Similar results were predicted for the influence of CT on BA, i.e., Cases 1 and 2 showed strong neighbor dependence of their interaction, but Case 3 did not. Interestingly, the influence of CT on PT was predicted to be membership dependent for all cases: the effect of CT on PT was negative in the quaternary culture, but the same in binary and ternary cultures was neutral in Cases 1 and 2, while positive in Case 3. We quantified modulation of interactions across binary and complex communities for Cases 1 to 3 (Figure 6). In all cases, we were able to predict considerable shifts in interspecies interactions across binary, ternary and quaternary communities.

## Discussion

The MIIA is a new concept of network inference that uniquely accounts for neighbor-dependent interactions in microbial communities. The MIIA (both original and scaled formulations) evaluates the effect of one species on another based on a simple analysis of population data. That is, ‘species 1’ is interpreted as playing a positive (or negative) role on the growth of ‘species 2,’ if ‘species 2’ increases (or decreases) its population density in the presence of ‘species 1.’ This general interpretation of species abundance data has been commonly used in the literature to understand microbial interactions (e.g., Paine, 1992; Kato et al., 2008). Equations (1) and (3) are an intuitive representation of that rationale, while mathematically derivable from a gLV model in steady state as shown in the original MIIA paper by Song et al. (2019). As an advantage, the MIIA uses only one time point at the end of growth (i.e., in the stagnant phase) without requesting the temporal profiles of species abundances to strictly follow gLV dynamics *in toto*.

While the original development was tested in ideal conditions where all species can grow solely as well as with partners, we proposed a new formulation to handle more realistic, complex systems by relieving that assumption. Despite data limitation, a synergistic combination of model scaling, parameter sensitivity analysis, and data coupling enabled predicting virtually all interaction networks across different memberships. This development not only expands the scope of prediction, but also contributes to creating a new understanding of interspecies interplay in a community.

To handle unidentifiable binary interaction coefficients, we parameterized them to perform sensitivity analyses (i.e., to examine how sensitively inference outputs change against their variations) and determine ranges or values based on additional experimental evidence. While additional knowledge employed in this work was based on traditional data analysis, this challenge can be more effectively overcome by integrating advanced experimental data including high-throughput multi-omics profiles (Franzosa et al., 2015), stable isotope labeling (Dumont and Murrell, 2005; Kanissery et al., 2018; Sun et al., 2018), imaging and probing technologies (He et al., 2010; Darch and Koley, 2018; Darch et al., 2018) and others.

When no additional data is available to determine unidentifiable binary parameters, we suggest assigning zeros as default values. While this initial assignment may not be fully generalizable, it can still serve as reference for sensitivity analysis. Interestingly, even without parameter tuning, our initial prediction using these default parameters were quite consistent with the interaction network experimentally determined from Kato et al. (2008). This was surprising because we only used a partial set of population data for this inference, while Kato et al. (2008) had to perform comprehensive experimental analyses to arrive at the same conclusion.

The sensitivity analysis not only allows to determine the robustness of predicted interactions, but also provides interesting biological insights into the effect of neighbors in microbial communities. Analysis of the entire results in Figure 4 led us to obtain the following two findings: (1) the correlations between the assumed $b_{i,j}^{B}$’s and the predicted $b_{i,j}^{C}$’s (where the superscript C = ternary or quaternary) are positive (as shown by thicker lines in Figures 4A–D) and (2) the correlations between assumed $b_{i,j}^{B}$’s and predicted $b_{i,k\neq j}^{C}$’s are negative (as shown by thinner lines). While magnitudes vary among cases, all correlations between binary and complex interaction coefficients followed these two patterns. The first rule is related to the minimal adjustment hypothesis of MIIA because it means that if the value of $b_{i,j}^{B}$ increases (decreases), the value of $b_{i,j}^{C}$ will also increase (decrease) and that the gap between those is adjusted to be toward being minimal. The second rule implies that there may exist trade-offs in alterations of interspecies interaction when the transition occurs from binary to complex communities. This implies that for example, in a ternary community, if the effect of species *j* on *i* is shifted in a positive/negative direction in the presence of species *k*, the effect of species *k* on *i* is shifted in a negative/positive direction in the presence of species *j*. This trade-off may be one of the mechanisms that potentially contribute to the formation of stable communities because this indicates that species can continue to exist in a community due to such compensatory effects among neighbor species.

The s-MIIA proposed in this work is a complementary method of the original formulation, rather than its replacement. The scaled method extends the application to a wider range of systems, but its prediction is limited to ‘relative’ changes in interactions. By contrast, the original MIIA provides absolute values of interaction coefficients, but cannot effectively handle ecological communities containing member species that are interdependent for growth. Such complementary predictions make their integration synergistic. Integration of the original and scaled MIIA makes the prediction more convincing and is extremely useful to understand interaction principles in diverse ecological perspectives, including the membership-dependent interactions. The predictive capacity of MIIA can solely allow us to identify the interaction coefficients on the complex consortia and its changes in terms of the introduction of new member species. In addition, MIIA may be able to provide an intriguing initial set of coefficients to incorporate with more computationally rigorous inference methods.

Modulation of interspecies interactions by a third-party species is a fundamental subject widely studied in community ecology (Chamberlain et al., 2014). This knowledge is particularly relevant not only for studying the effect of species invasion in microbial communities in environment, but also for designing synthetic consortia by partnering platform organisms with new additional species (Lindemann et al., 2016; Song, 2018; Song et al., 2018). Furthermore, in view of a current innovative research trend that utilizes compositionally simple model consortia for understanding interactions in complex communities (Haruta and Yamamoto, 2018), prediction of the impact of additional species on existing interactions is a critical step to bridge the gap between the two systems.

## Data Availability Statement

All datasets generated for this study are included in the article/Supplementary Material.

## Author Contributions

J-YL, SH, and H-SS conceived the project. All authors contributed to design the research. J-YL and H-SS developed the method, implemented the algorithm, and analyzed the results. J-YL, SH, and H-SS drafted out the manuscript, which was edited by HB, SL, JF, D-YL, and SK. All authors read and approved the final manuscript.

## Conflict of Interest

The authors declare that the research was conducted in the absence of any commercial or financial relationships that could be construed as a potential conflict of interest.

## Supplementary Material

The source code written in Matlab is available at: https://github.com/hyunseobsong/miia

FIGURE S1 Performance of s-MIIA against measurement error: The effect of measurement error on the predictive capability of s-MIIA was tested for a five-member community using simulated data generated by a generalized Lotka-Volterra (gLV) model as described in Song et al. (2019). For the purpose of comparison with the original MIIA, the gLV parameter values used in generating simulated data are the same as those used in Song et al. (2019). Noisy data were generated based on a multiplicative lognormal noise: $x_{n\,o\,i\,s\,e}=x\,\exp\left[N\,\left(0,\delta_{n\,o\,i\,s\,e}^{A,B}\right)\right]$ for species abundances in axenic and binary cultures; $x_{n\,o\,i\,s\,e}=x\,\exp\left[N\,\left(0,\delta_{n\,o\,i\,s\,e}^{C}\right)\right]$ for species abundances in complex cultures where *x* is the species abundance obtained from the steady-state gLV model without noise; $\delta_{n\,o\,i\,s\,e}^{A,B}$ and $\delta_{n\,o\,i\,s\,e}^{C}$ denote metrics for the level of introduced noise in axenic/binary and complex cultures, respectively. The results show that the prediction of s-MIIA is robust over a reasonable range of noise level (when $\delta_{n\,o\,i\,s\,e}^{C}\leq 0.1$ and $\delta_{n\,o\,i\,s\,e}^{A,B}\leq 0.01$), which is comparable to the performance of the original MIIA demonstrated in Song et al. (2019). *S*_*C*_ is cosine similarity; *b*^*C*^is the true interaction coefficient in a complex community; ${\hat{b}}_{n\,o\,i\,s\,e}^{C}$is the interaction coefficient in a complex community estimated under the noise effect. **(A)** $\delta_{n\,o\,i\,s\,e}^{A,B}$ = 0.0001, **(B)** $\delta_{n\,o\,i\,s\,e}^{A,B}$ = 0.001, **(C)** $\delta_{n\,o\,i\,s\,e}^{A,B}$ = 0.01, and **(D)** $\delta_{n\,o\,i\,s\,e}^{A,B}$ = 0.1.
