# Supplementary figures and images for: Prediction of Neighbor-Dependent Microbial Interactions From Limited Population Data
Source: Front Microbiol. 2020 Jan 21;10:3049. doi: 10.3389/fmicb.2019.03049 (PMC6985286; doi:10.3389/fmicb.2019.03049)

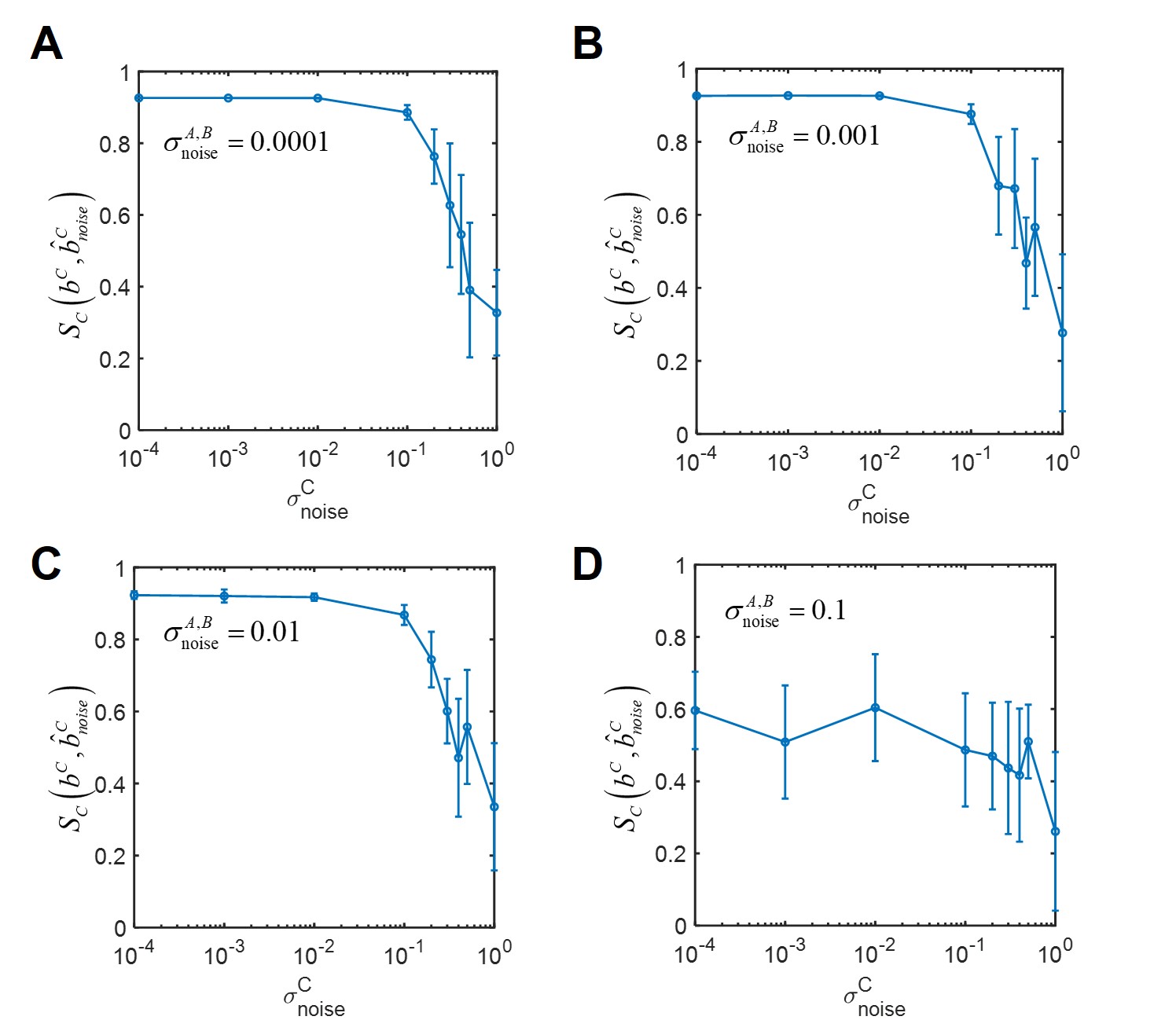

Supplement: FIGURE S1 — Performance of s-MIIA against measurement error: The effect of measurement error on the predictive capability of s-MIIA was tested for a five-member community using simulated data generated by a generalized Lotka-Volterra (gLV) model as described in Song et al. (2019). For the purpose of comparison with the original MIIA, the gLV parameter values used in generating simulated data are the same as those used in Song et al. (2019). Noisy data were generated based on a multiplicative lognormal noise: xn⁢o⁢i⁢s⁢e=x⁢exp⁡[N⁢(0,δn⁢o⁢i⁢s⁢eA,B)] for species abundances in axenic and binary cultures; xn⁢o⁢i⁢s⁢e=x⁢exp⁡[N⁢(0,δn⁢o⁢i⁢s⁢eC)] for species abundances in complex cultures where x is the species abundance obtained from the steady-state gLV model without noise; δn⁢o⁢i⁢s⁢eA,B and δn⁢o⁢i⁢s⁢eC denote metrics for the level of introduced noise in axenic/binary and complex cultures, respectively. The results show that the prediction of s-MIIA is robust over a reasonable range of noise level (when δn⁢o⁢i⁢s⁢eC≤0.1 and δn⁢o⁢i⁢s⁢eA,B≤0.01), which is comparable to the performance of the original MIIA demonstrated in Song et al. (2019). SC is cosine similarity; bCis the true interaction coefficient in a complex community; b^n⁢o⁢i⁢s⁢eCis the interaction coefficient in a complex community estimated under the noise effect. (A) δn⁢o⁢i⁢s⁢eA,B = 0.0001, (B) δn⁢o⁢i⁢s⁢eA,B = 0.001, (C) δn⁢o⁢i⁢s⁢eA,B = 0.01, and (D) δn⁢o⁢i⁢s⁢eA,B = 0.1. [file Image_1.JPEG]
